# Anti-coagulation for COVID-19 treatment: both anti-thrombotic and anti-inflammatory?

**DOI:** 10.1007/s11239-020-02212-6

**Published:** 2020-07-06

**Authors:** Vera Paar, Bernhard Wernly, Zhichao Zhou, Lukas J. Motloch, Uta C. Hoppe, Alexander Egle, Michael Lichtenauer

**Affiliations:** 1 Department of Internal Medicine II, Division of Cardiology, Paracelsus Medical University of Salzburg, Salzburg, Austria; 2 Department of Medicine, Division of Cardiology, Karolinska Institutet, Karolinska University Hospital, Stockholm, Sweden; 3 Department of Internal Medicine III, Hematology, Medical Oncology, Hemostaseology, Rheumatology and Infectious Diseases, Oncologic Center, Paracelsus Medical University of Salzburg, Salzburg, Austria

**Keywords:** SARS-CoV-2, Inflammation, Coagulation, Thrombosis, Fibrin, Cytokines, Chemokines

## Abstract

Severe acute respiratory syndrome coronavirus-2 (SARS-CoV-2) infection has been linked to a higher risk of mortality compared to influenza, which is mainly due to severe secondary diseases, such as acute respiratory distress syndrome (ARDS). In turn, ARDS is characterized by an acute inflammation and an excessive activity of the coagulation cascade, rising the vulnerability for venous thromboembolic events. In order to investigate the relation of inflammation and the influence of coagulation factors on their release, human peripheral mononuclear blood cells (PBMCs) were treated with autologous serum, heparinized plasma and different doses of fibrin. Thereafter, the concentration of pro-inflammatory cytokines and chemokines in the secretome of PBMCs was measured by enzyme-linked immunosorbent assay. Our analyses revealed autologous serum to significantly increase the secretion of cytokines and chemokines after 24 h of incubation time. Furthermore, the addition of fibrin markedly increased the secretion of cytokines and chemokines by PBMCs in a dose-dependent manner. Consequently, in accordance with previous studies, our study outlines that anti-coagulation may constitute a promising tool for the treatment of SARS-CoV-2, reducing both, the cytokine storm, as well as the risk for thrombotic complications.

## Highlights

- The novel coronavirus-2 (SARS-CoV-2) was already shown to induce a systemic inflammatory response, which is accompanied by an excessive release of pro-inflammatory cytokines.
- Furthermore, COVID-19 is commonly accompanied by hemodynamic abnormalities, such as an increased coagulation activity, increasing the risk for venous thromboembolic events.
- In our short communication we focus on the relation of cytokines’ and chemokines’ release and the influence of coagulation factors on the inflammatory response of peripheral mononuclear blood cells (PBMCs).
- After 24 h of incubation time, 20% serum was the main trigger for the release of several cytokines and chemokines in comparison to heparinized plasma. Furthermore, it was shown that the addition of fibrin to the PBMCs resulted in a significant increase of the cytokines and chemokines measured in a dose-dependent manner.
- Anti-coagulation could constitute a potential tool to handle and treat the circle between inflammation and thrombosis in COVID-19, reducing a profound cytokine storm and thrombotic complications.

In December 2019, the new coronavirus severe acute respiratory syndrome coronavirus-2 (SARS-CoV-2) emerged, causing coronavirus disease 2019 (COVID-19). SARS-CoV-2 was found to infect lung alveolar cells, cardiac myocytes, vascular epithelial and endothelial cells, as well as inflammatory cells including macrophages, monocytes and lymphocytes [1, 2]. This phenomenon is primarily due to the virus’ portal of entry, the angiotensin-converting enzyme 2 (ACE2) receptor, which is highly expressed in vascular tissues [3]. Consequently, endothelial injury is a crucial indicator of SARS-CoV-2 infection [2]. Clinically, the disease manifests with symptoms, including fever, cough, dyspnea, and myalgia. The infection mortality rate is a subject to debate, but likely higher than for seasonal influenza [4]. The higher risk of mortality is primarily due to the risk for severe illnesses, such as systemic inflammatory response syndrome, septic shock, multi-organ involvement, and most notably acute respiratory distress syndrome (ARDS) [5]. There is also increasing evidence that COVID-19 differs from “classic” ARDS with regards to respiratory characteristics and optimal ventilatory management [6].

Generally, acute inflammation, intravascular coagulation and platelet activation characterize ARDS. In COVID-19 patients, excessive cytokine release and cytokine storm have been reported in response to SARS-CoV-2 infection. In particular, vast amounts of the pro-inflammatory cytokine interleukin-6 (IL-6) are released; although whether IL-6 is foremost a marker for the severe illness or the disease’s mediator itself is still unknown yet, its levels in circulation seem to correlate with disease severity and the propensity for a pro-coagulant status. In addition to IL-6, also higher levels of IL-1β, IL-2, IL-4, IL-7, IL-10, interferon-gamma (IFN-γ), IF-γ-induced protein 10 (IP-10), tumor necrosis factor-alpha (TNF-α), granulocyte-colony stimulating factor, macrophage inflammatory protein 1-alpha, and myocyte chemotactic protein (MCP-1), as well as C-reactive protein (CRP), and d-dimer have been found in circulation. This cytokine storm may also seem to correlate with the severity of disease or mortality [7]. Due to the lack of not only an appropriate vaccine, but also effective antiviral treatment to SARS-CoV-2, anti-cytokine therapies targeting IL-6 or IL-1 have been proposed to serve as a potential therapy against the ongoing cytokine storm [5, 8]. The IL-6 inhibitor tocilizumab is currently under investigation to serve as a treatment option for COVID-19 [5, 8].

**Fig. 1:**
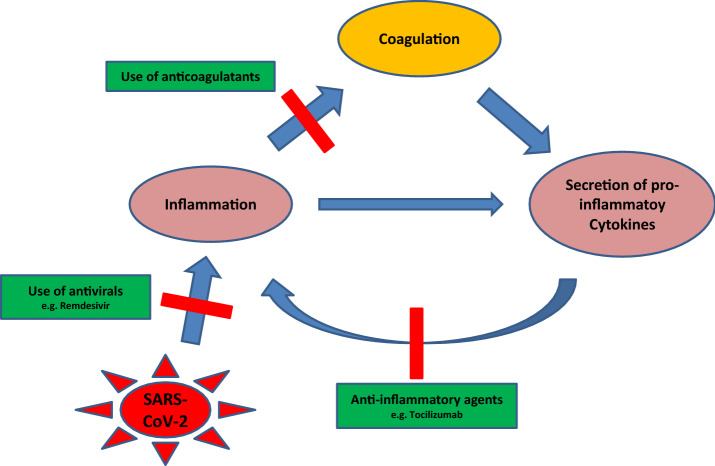
Schematic representation of the interactions between coagulation and inflammation in response to SARS-CoV-2 infection, including the secretion of pro-inflammatory cytokines

Furthermore, recent studies revealed that SARS-CoV-2 infections are commonly accompanied by hemostatic abnormalities, such as elevated coagulation activity, leading to disseminated intravascular coagulation (DIC) [9]. Complement activation is one initiating pathway responsible for the innate immune responses, that promotes inflammation and often defends against bacteria and virus infections. Furthermore, the complement system and the coagulation cascade are closely linked, which may provoke hypercoagulability and thrombosis. Excessive activation of the complement system is therefore associated with several pathologic events, such as thrombotic microangiopathy and organ dysfunction (Fig. 1). It has already been described that patients with SARS-CoV-2 infections exhibit a higher risk for venous thromboembolic events, including pulmonary embolism. The clinical parameters of patients suffering from severe COVID-19 correlate with an excessive complement activity: high lactate dehydrogenase, d-dimer, and bilirubin. Concerning this, increased levels of d-dimer were shown to serve as a particularly important marker for coagulopathy [10] and pulmonary embolism [11]. Additionally, an in vitro study in which Vero cells were infected with a SARS-associated CoV has outlined the potential of heparin to inhibit the infection by 50% [12]; in vivo, heparin has already proven to significantly lower the rate of mortality due to SARS-CoV-2 infection [13]. Consequently, there is evidence suggesting a link between the coagulation pathway and the inflammatory response.

To prove this assumption, we performed an in vitro study to investigate the potential of an activated coagulation cascade or clotting factors to trigger the expression and release of pro-inflammatory cytokines. Human peripheral mononuclear blood cells (PBMCs) from healthy volunteers (n = 7) were treated with either autologous serum or heparinized plasma, respectively. As in serum the coagulation cascade has already been progressed to a final state, the addition of autologous serum used to clarify the influence of the coagulation factors released into serum on the PBMCs. On the contrary, in heparinized plasma the coagulation cascade is inhibited due to the presence of heparin, presenting a hypocoagulable environment. In addition to that, the different doses of the coagulation factor fibrin (Sigma-Aldrich, USA) were used to unveil the effect of this specific coagulation factor on cytokine expression and secretion. Cells were incubated at 37 °C and 4% CO_2_ at the different treatment conditions (negative control, autologous serum, autologous heparinized plasma, or fibrin) for 0 h (h), 4 h, or 24 h, respectively. Supernatant cytokine and chemokine concentrations were measured using commercially available enzyme-linked immunosorbent assay kits purchased from R&D Systems (Minneapolis, MN, USA): human IL-1 beta/IL-1F2 DuoSet, human IL-6 DuoSet, human IL-8 /CXCL8 DuoSet, human CCL2/MCP-1 DuoSet, and human TNF-alpha DuoSet, and human IL-1ra. The results were calculated in GraphPad PRISM software (GraphPad-Software, La Jolla, CA, USA). Due to non-gaussian approximation, the results are given as median ± interquartile range (IQR).

**Fig. 2:**
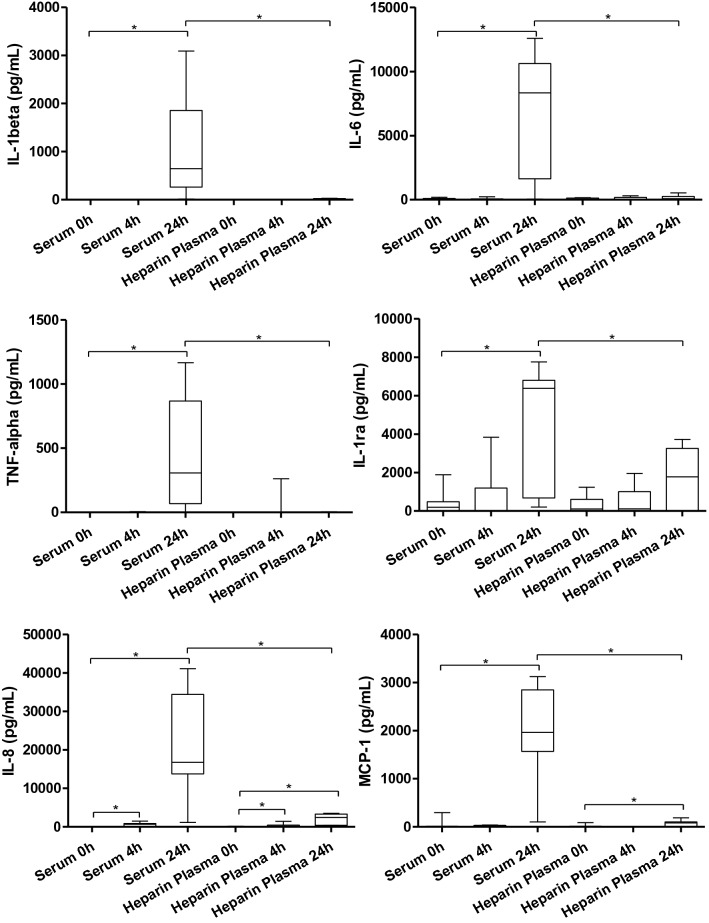
Supernatant protein levels following the treatment with 20% autologous serum or heparinized plasma, respectively, at baseline (0 h), 4 h, and 24 h after PBMC treatment. *p < 0.05; **p < 0.01; ***p < 0.001. *h* Hours; *IL-1β/-6/-8* Interleukin-1beta/-6/-8; *IL-1ra* Interleukin-1 receptor antagonist; *MCP-1* Monocyte chemoattractant protein 1; *pg/ml* Picograms per milliliters; *TNF-α* Tumor necrosis factor alpha

Our results revealed that the treatment of PBMCs with 20% autologous serum for 24 h, in comparison to heparinized plasma, led to the greatest inflammatory response, outlined by the significant alteration of inflammatory cytokines and chemokines in the secretome. In particular, the pro-inflammatory cytokines IL-1β, and IL-6 were significantly elevated in the PBMC’s supernatant, rising from 0.0 ± 0.0 pg/ml at baseline to 664.6 ± 1591.7 pg/ml (p = 0.02) after 24 h for IL-1β, and an IL-6 concentration from 82.4 ± 41.9 pg/ml at baseline to 8331.0 ± 8986.0 pg/ml (p = 0.03). Also, TNF-α, and IL-1ra were significantly altered by the treatment of PBMCs with autologous serum. Moreover, the chemokine IL-8 increased markedly from 66.6 ± 66.1 pg/ml at baseline to 16,776.0 ± 20,665.0 pg/ml at 24 h (p = 0.02), as it is the case for MCP-1 (0.0 ± 11.0 pg/ml at baseline to 1965.0 ± 1,280.0 pg/ml after 24 h, p = 0.016). Although the treatment of PBMCs by heparinized plasma resulted in a significant increment of supernatant IL-8 and MCP-1 levels, the increase was not as high as it is the case for autologous serum. Furthermore, all cytokines and chemokines measured were markedly higher for serum than for heparinized plasma (after 24 h; see Fig. 2).

**Fig. 3:**
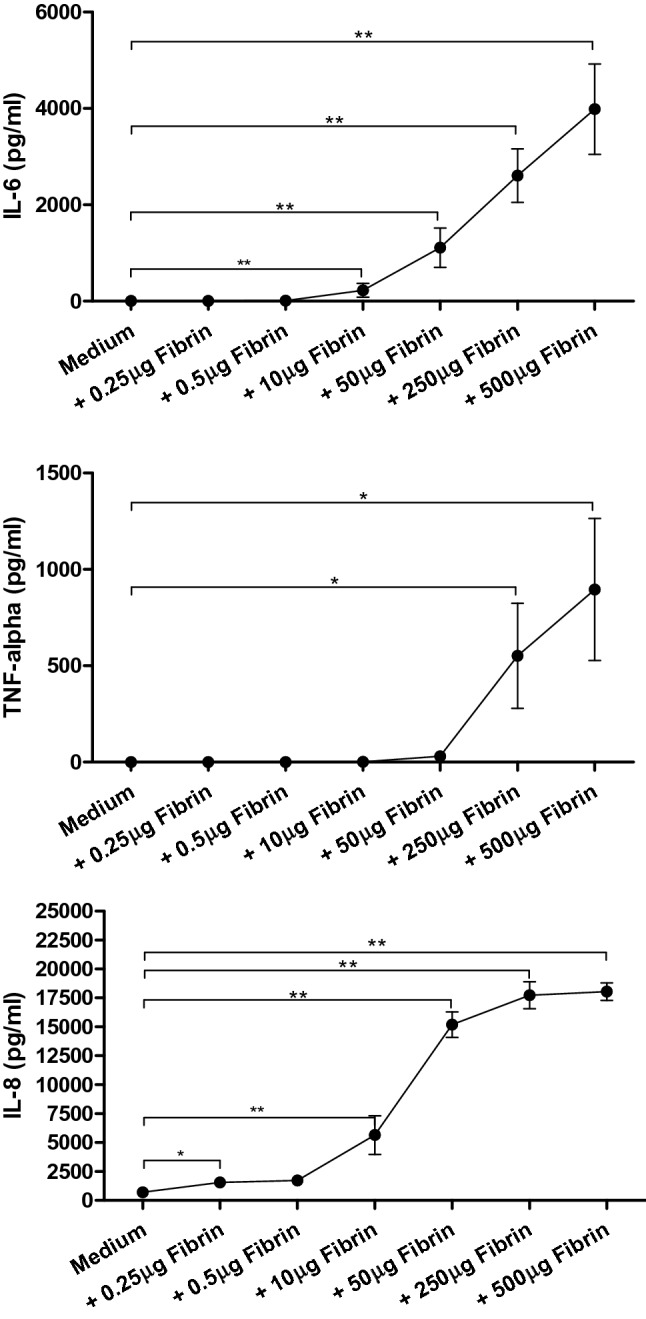
Supernatant protein levels of PBMCs following the treatment with different concentrations of fibrin. *p < 0.05; **p < 0.01; ***p < 0.001. *IL-6/-8* Interleukin-6/-8; *TNF-α* Tumor necrosis factor alpha; *μg* Micrograms; *pg/ml* Picograms per milliliters

We analyzed the secretome of PBMCs treated with rising concentrations of fibrin (0.25 µg, 0.5 µg, 10 µg, 50 µg, 250 µg, and 500 µg) for 24 h. Our data unveiled a significant rise of IL-6, TNF-α, and IL-8 in a concentration-dependent manner; the treatment of PBMCs with 500 µg fibrin resulted in the greatest cytokine and chemokine values: For instance, IL-6 supernatant levels rose from 10.45 ± 15.3 pg/ml at baseline to 4000.0 ± 3,461.0 pg/ml (p = 0.008) due to the treatment with 500 µg fibrin. In addition to that, the chemokine IL-8 was significantly altered by the treatment with fibrin (from 640.4 ± 907.3.9 pg/ml to 17,475.0 ± 3272.0 pg/ml, p = 0.01, see Fig. 3). These in vitro results confirm the association of coagulation and the inflammatory response of the immune system.

Our data support the findings of Yang et al. [14] where plasma samples of patients infected with SARS-CoV-2 were examined and outlined an increase of 14 cytokines triggering inflammation in response to COVID-19. There are many other evidence that the activation of coagulation has a marked impact on the cytokine-storm associated with the infection. As recently reviewed in Bikdeli et al. [9], among others, there are several hemostatic changes in response to SARS-CoV-2 infection, including mild thrombocytopenia and increased d-dimer levels. Furthermore, some case studies indicate an association between the severity of disease and a potential prolongation of prothrombin time and thrombin time, as well as a shortened activated partial thromboplastin time. Concerning this, our study confirms that coagulation may increase the inflammatory response, eventually resulting in a feedback loop, further triggering the hypercoagulable state. Although, our data does not outline the reverse progress that inflammation may serve as a trigger for a hypercoagulable state, it is already known that e.g. sepsis may be complicated by DIC. Supporting our data and speculations, a recent study by Patel et al. addressed the expression of inflammatory markers in sepsis an DIC and could unveil a positive correlation of IL-6, IL-8, IL-10, and TNF-α with DIC score [15].

In addition to that, severe cases of COVID-19 exhibit an increased risk of developing venous thromboembolism (VTE). A Chinese study reported that 40% of COVID-19 patients were highly prone to VTE [16]. Also, Danzi et al. [10] reported a case study that a woman infected with SARS-CoV-2 was hospitalized due to severe bilateral pneumonia. CT scans have unveiled a severe pulmonary embolism, although no predisposing factors for VTE were known. In addition, patients with severe pneumonia due to COVID-19 had a higher platelet count, contributing to the increased risk of thrombosis [13]. In a small case series a relation between COVID-19 and large-vessel stroke was also proposed [17].

Based on available in vitro data and first clinical impressions, anti-coagulation could be a cornerstone in successfully managing COVID-19 [9, 13, 18]. Besides preventing thrombotic complications, anti-coagulation could mitigate the vicious circle between inflammation and thrombosis in COVID-19. Therapeutic anti-coagulation might be warranted in critically sick COVID-19 patients. For non-severely ill COVID-19 patients, preventive anti-coagulation could reduce thrombotic complications and have the potential to help minimize the risk for full-blown cytokine storm. Although COVID-19 patients could benefit from the treatment with anti-coagulants, it is not yet proven that their usage would also reduce the inflammatory response due to SARS-CoV2 infection. As far as the safety of the treatment with oral anticoagulants is concerned, the risk for advanced bleeding of COVID-19 patients has to be considered.
